# Vemurafenib-resistant BRAF selects alternative branch points different from its wild-type BRAF in intron 8 for RNA splicing

**DOI:** 10.1186/s13578-015-0061-7

**Published:** 2015-12-21

**Authors:** Masahiko Ajiro, Zhi-Ming Zheng

**Affiliations:** Tumor Virus RNA Biology Section, Gene Regulation and Chromosome Biology Laboratory, National Cancer Institute, National Institutes of Health, Frederick, MD 21702 USA

**Keywords:** BRAF, RNA splicing, Branch point mapping, Lariat RT-PCR, Vemurafenib-resistance

## Abstract

One mechanism of resistance of the melanoma-associated BRAF kinase to its small molecule inhibitor vemurafenib is by point mutations in its intron 8 resulting in exons 4–8 skipping. In this report, we carried out in vitro BRAF RNA splicing assays and lariat RT-PCR to map the intron 8 branch points in wild-type and BRAF mutants. We identify multiple branch points (BP) in intron 8 of both wild-type (wt) and vemurafenib-resistant BRAF RNA. In wt BRAF, BPs are located at -29A, -28A and -26A, whereas in a vemurafenib-resistant BRAF splicing mutant, BPs map to -22A, -18A and -15A, proximal to the intron 8 3′ splice site. This finding of a distal-to-proximal shift of the branch point sequence in BRAF splicing in response to point-mutations in intron 8 provides insight into the regulation of BRAF alternative splicing upon vemurafenib resistance.

**Electronic supplementary material:**

The online version of this article (doi:10.1186/s13578-015-0061-7) contains supplementary material, which is available to authorized users.

## Background

BRAF proto-oncogene encodes a serine/threonine kinase regulator of the MAP kinase pathway, and activating BRAF mutations are found in 40–60 % of melanoma, with 90 % of them containing the V600E mutation [1, 2]. Vemurafenib, a potent inhibitor of (V600E) BRAF in melanoma cells, is currently in clinical use [3–5]. However, patients treated with vemurafenib develop resistance by activation of alternative signaling pathways [6–9] or by inducing alternative splicing of BRAF to exclude the RAS-binding domain encoded by exons 3–5 [10]. The vemurafenib-resistant melanoma cell line C3 SKMEL-239 produces BRAF exon 3^9 splicing and contains two intronic point-mutations at positions -435 (C-to-A) and -51 (C-to-G) from the BRAF intron 8 3′ splice site. In a minigene system the -51 mutation, located in the computationally predicted branch point (BP), was found to be sufficient to recapitulate BRAF exon 3^9 splicing [10, 11].

Each intron of eukaryotic primary RNA transcripts (pre-mRNAs) has a 5′ splice site with a GU dinucleotide and a 3′ splice site with an AG dinucleotide. The 3′ splice site also contains a BP in a 7-nt or 5-nt branch point sequence (BPS) and a run of 15–40 pyrimidines (usually Us), called polypyrimidine tract (PPT), between the BPS and the 3′ end AG dinucleotide. Defining the exon–intron boundary in pre-mRNA splicing is the first step in the accurate recognition of an intron 5′ splice site by U1 snRNA, of BPS by U2 snRNA, and of a 3′ splice site by U2AF (U2 auxiliary factors) modulated by many cellular splicing factors [12–14]. These recognition steps are followed by two transesterification reactions during spliceosome assembly. In this two-step biochemical reaction, an OH group of the BP adenosine within the BPS performs a nucleophilic attack on a phosphodiester bond of the intron-5′ exon junction, resulting in the first step in the 5′ exon being cleaved off and forming a lariat intermediate by a branching reaction of the intron 5′ end G to the BP adenosine via a 5′-to-2′ phosphodiester link. The second step is to cleave the intron from the lariat intermediate by another nucleophilic attack of the OH group from the cleaved 5′ exon on a phosphodiester bond of the intron-3′ exon junction and join the cleaved 5′ exon to the cleaved 3′ exon. Thus, if an intron 5′ and 3′ splice sites are of consensus sequence, they sequentially bind three different splicing factors in order to assemble the spliceosome. However, the splice sites in higher eukaryotes are usually not well conserved and binding of splicing factors to pre-mRNAs with non-consensus sequence is often inefficient. In addition, pre-mRNA splicing is subject to regulation by other intronic or exonic *cis*-elements, intronic splicing enhancers (ISE) or silencers (ISS) and exonic splicing enhancer (ESE) or silencer (ESS), often located at a distance. The combination of the strength of the various *cis*-regulatory elements and the local availability of splicing factors determines alternative splicing outcome [13, 14].

In this report, we experimentally mapped the BPS in BRAF intron 8 that controls the constitutive RNA splicing of wild-type (wt) BRAF exon 8^9 and discovered an alternative BPS in the intron 8 of a vemurafenb-resistant mutant (mt) BRAF pre-mRNA.

## Result and discussion

### BRAF intron 3 and intron 8 are suboptimal

**Fig. 1:**
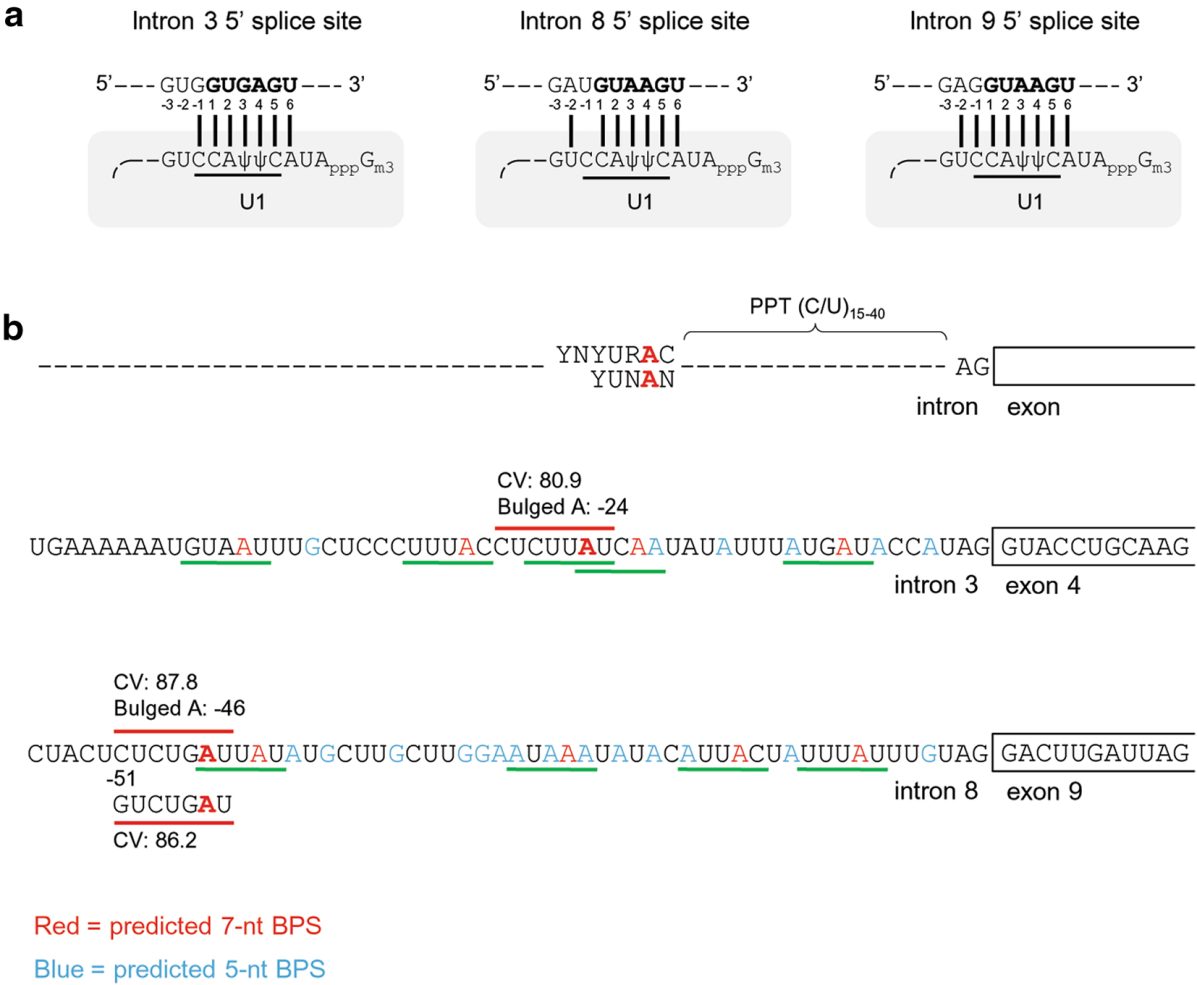
Suboptimal features of the BRAF intron 3 and intron 8. **a** The sequences of indicated 5′ splice sites in base-pairing with the 5′ end of U1 snRNA. The *lines* below the U1 sequences indicate six most important U1 5′end nucleotides (core) for base-pairing with each 5′ splice site during 5′ splice site recognition. The intron 5′ end sequences are bolded with the intron 5 end G as position 1 and the exon 3′ end nucleotide immediately upstream of the intron 5′ end G as position -1. **b** The sequences of intron 3 and intron 8 3′ splice sites with predicted 7-nt (*red lines*) and 5-nt BPS (*green lines*). The mammalian consensus 7-nt BPS sequence YNYURAC [23, 24] or 5-nt YUNAN [25–27] are shown for comparison. *Red color A* in the predicted 7-nt BPS and in the predicted 5-nt BPS indicates the putative BP adenosine A. Conservation value (CV) for each predicted 7-nt BPS is calculated by using Human Splice Finder (http://www.umd.be/HSF/), with indicated distance of the bulged A from the downstream 3′ splice site. Purines in the putative PPT are labeled in *light blue or red*

Both annotated BRAF intron 3 and intron 8 are large, with a size of ~25.6 kb for the intron 3 and 6.7 kb for intron 8. To understand what might contribute to the observed alternative exon 3^9 splicing of vemurafenib-resistant BRAF RNA, we analyzed sequence structures of the 5′ and 3′ splice sites in BRAF introns 3 and 8 and also the 5′ splice site of BRAF intron 9 considering that exon definition may play an important role in defining an upstream 3′ splice site [13, 15, 16]. Recognition of an intron 5′ splice site by U1 snRNA requires the 5′-terminal 11 nts of the U1 snRNA to base-pair directly with the 5′ splice site [17]. We found that all three analyzed 5′ splice sites have consensus sequence GURAGU [18, 19], but only intron 3 and intron 9 5′ splice sites can base-pair fully to the six core nucleotides of the U1 snRNA 5′ end, whereas the intron 8 5′ splice site is missing a nucleotide at position -1 upstream of the 5 splice site (Fig. 1a). The inability of the U1 snRNA 5′ core nucleotide to fully base-pair with the 5′ splice site affects U1 snRNA binding and decreases splice site strength [20–22].

In general, a consensus 3′ splice site is composed of three critical elements: BPS, PPT (usually with a stretch of U residues), and an AG dinucleotide at the 3′ end of the intron. Mammalian consensus BPSs are YNYURAC [23, 24] or YUNAN, [25–27] with 90 % of BPSs occurring within 19–37 (median 25) nucleotides upstream of the 3′ AG dinucleotides and 78 % of the BP nucleotides within a BPS being an adenosine [27]. Analysis of the intron 3 and intron 8 3′ splice sites using Human Splice Finder (http://www.umd.be/HSF/) [28] revealed that both introns bear a non-consensus 7-nt BPS within the distance range in intron 3, but further upstream (46 nts) in intron 8. The intron 8 3′ splice site is also predicted to have multiple non-consensus 5-nt BPSs within the distance range to its 3′ AG dinucleotide (Fig. 1b). Moreover, both introns have a weak PPT interspersed by purines with runs of uridines no longer than three. Altogether, the weak nature of these 3′ splice sites would subject them to regulation by RNA *cis*-elements or *trans*-acting factors.

### Reconstitution of wt exon 8^9 and mt exon 3^9 splicing of BRAF in vitro

**Fig. 2:**
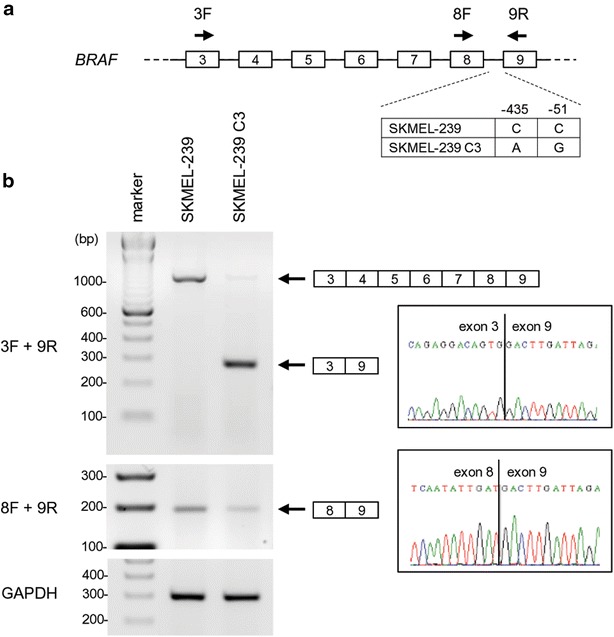
Activation of BRAF exon 3^9 splicing in C3 SKMEL-239 cells. **a** Diagrams showing primers used for detection of BRAF exon 8^9 or exon 3^9 RNA splicing by RT-PCR and point mutations in the intron 8. **b** RT-PCR detection of the constitutive BRAF exon 8^9 splicing in wt SKMEL-239 cells and the alternative BRAF exon 3^9 splicing in vemurafenib-resistant C3 SKMEL-239 cells. GAPDH RNA was used as a loading control. RT-PCR products were gel-purified and sequenced. The splicing junction of each product is shown on the *right chromatograms*

**Fig. 3:**
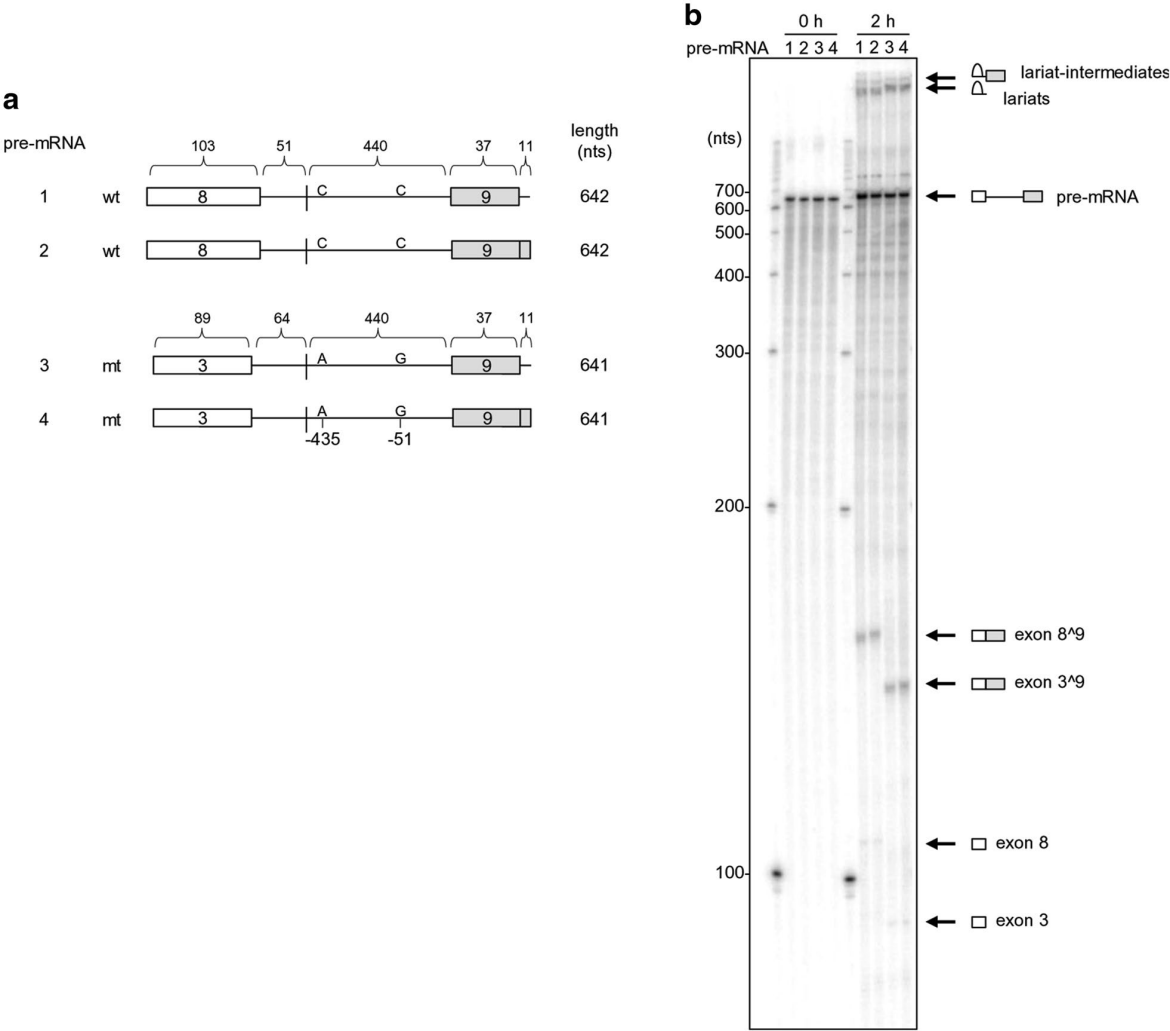
Reconstitution of BRAF RNA splicing for wt and mt BRAF pre-mRNAs. **a** Diagrams of BRAF wt and mt pre-mRNAs with a native 5′ splice site (an 11-nt U1-binding site) from the intron 9 (pre-mRNA 1 and 3) or a consensus 11-nt U1-binding site (pre-mRNA 2 or 4) used for in vitro RNA splicing assays, with the length of exons and a truncated intron indicated in nts for each pre-mRNA. Positions of mutated nts in the BRAF intron 8 found in vemurafenib-resistant cells are indicated. **b** In vitro splicing assay result for BRAF pre-mRNAs. Identities of individual splicing products in a splicing gel are shown on the *right*

When compared to the melanoma SKMEL-239 cells which are sensitive to vemurafenib treatment, the vemurafenib-resistant melanoma C3 SKMEL-239 cells harbor both -435 C-to-A and -51 C-to-G mutations within the BRAF intron 8 and exhibit activation of BRAF exon 3^9 splicing, leading to reduction of the constitutive BRAF exon 8^9 splicing of BRAF (Fig. 2a, b). The -51 mutation has been shown to be sufficient to induce exon 3^9 splicing in a BRAF minigene system [11]. To map the BPS directing exon 8^9 and exon 3^9 splicing of BRAF in vitro, and because the annotated BRAF intron 3 (~25.6 kb) and intron 8 (6.7 kb) are extremely large, we constructed a wt BRAF DNA template with a truncated intron 8 from SKMEL-239 cells and a mt BRAF DNA template with a chimeric intron 3 and intron 9 from C3 SKMEL-239 cells for generation of pre-mRNAs under a T7 promoter. Thus, the wt BRAF pre-mRNA transcribed in vitro had a truncated intron 8 from the middle of the intron and the mt BRAF pre-mRNA had a chimeric intron of which the intron 3 5′ splice site (64 nts) was fused with the intron 8 3′ splice site (440 nts) including the point mutations in the intron (Fig. 3a). The 3′ end of each BRAF pre-mRNA used in this assay also contained a native 5′ splice site (a U1-binding site) from intron 9 (Fig. 3a, pre-mRNAs 1 and 3, also Fig. 1a) or a consensus U1-binding site (Fig. 3a, pre-mRNAs 2 and 4) to promote RNA splicing efficiency [29]. In vitro RNA splicing was conducted in the presence of HeLa nuclear extract [30, 31]. This in vitro RNA splicing assay revealed that both wt and mt BRAF pre-mRNAs spliced equally efficiently in a 2 h reaction, with the expected sizes of splicing products (Fig. 3b) and accumulation of splicing lariats and lariat-intermediates from all four pre-mRNAs (Fig. 3b, top two bands). There was no difference in splicing efficiency among the BRAF pre-mRNAs with a consensus U1 binding site or a native U1 binding site from the intron 9 attached to the RNA 3′ end. Interestingly, we noticed that the lariats and lariat-intermediates derived from mt BRAF exon 3^9 splicing were running slower than that of wt BRAF exon 8^9 splicing in a 6 % denaturing PAGE gel (Fig. 3b). Although the intron of mt BRAF pre-mRNAs is 13 nts longer than that of the wt BRAF pre-mRNAs, the observed slowly migrating lariats and lariat-intermediates derived from mt BRAF pre-mRNAs suggested that a larger loop in the mt lariats than the wt lariats was formed when a 5′–2′ phosphodiester branching reaction during mt RNA splicing occurred between the intron 5′ splice site GU and a BP nucleotide. These data indicate that the mt RNA might utilize a BPS closer to the intron 3′ splice site than the wt RNA.

### Identification of distinct sets of alternative BPs for wt and mt BRAF splicing by lariat RT-PCR

**Fig. 4:**
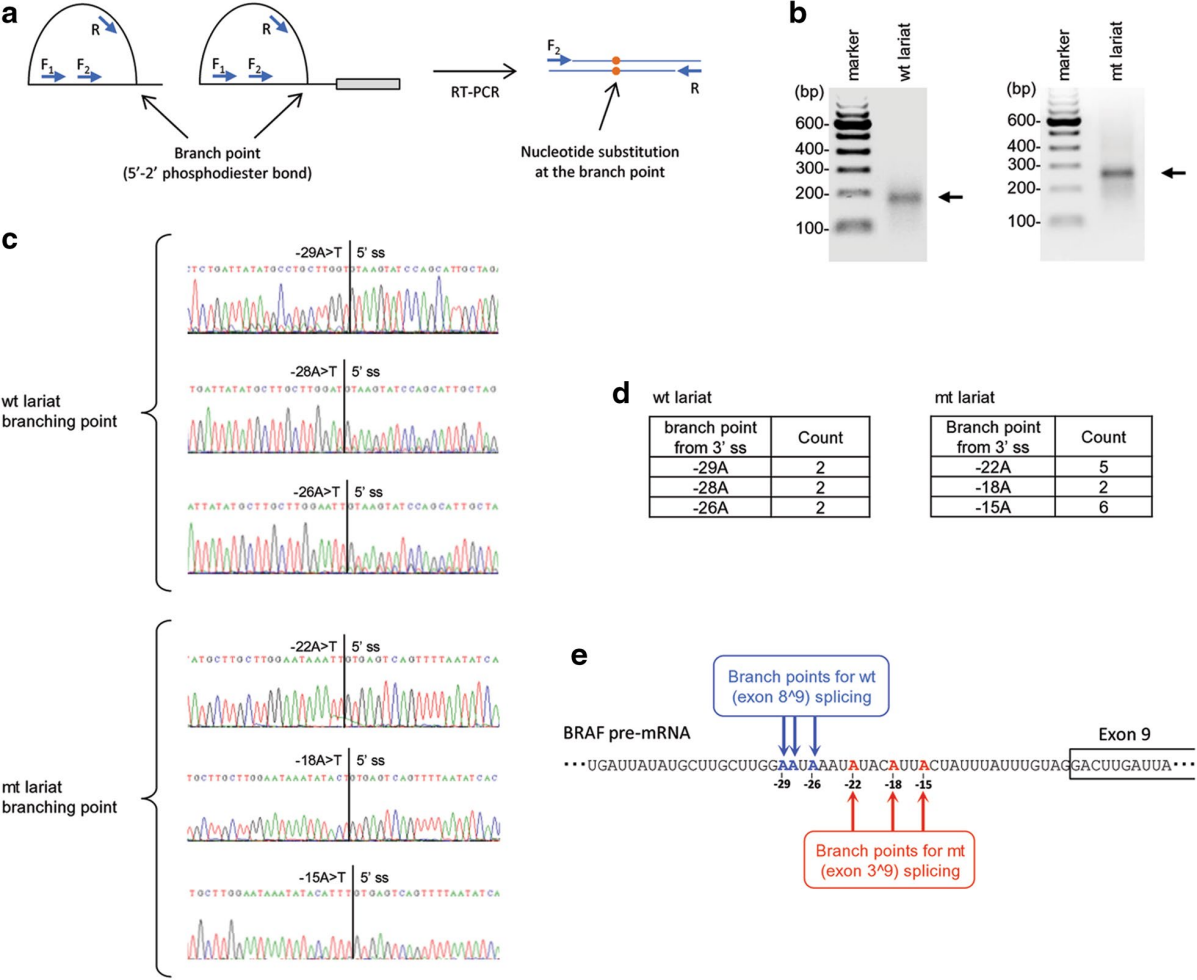
Distinct BPS usage of wt BRAF from mt BRAF RNA splicing identified by lariat RT-PCR. **a** Strategy of lariat RT-PCR for branch point mapping. Splicing lariats or lariat-intermediates from in vitro splicing assays were reverse-transcribed by a primer R. PCR amplification of the RT products was carried out by a primer set of F_1_ and R, and then nested by another primer set of F_2_ and R. **b** Lariat RT-PCR products (*arrows*) of wt (*left*) and mt (*right*) BRAF RNA splicing. **c** The mapped branch points from TA cloning and sequencing of the wt and mt BRAF lariats and lariat-intermediates. **d** Frequency of the mapped branch points from wt and mt BRAF splicing by lariat RT-PCR in combination with TA cloning and sequencing. **e** Illustration of the mapped branch points in the intron 8 used for RNA splicing of the wt and mt BRAF

Given the successful reconstitution of wt and mt BRAF splicing in vitro, we then performed lariat RT-PCR on the lariats and lariat-intermediates derived from BRAF pre-mRNAs 1 and 3. In the lariat RT-PCR, SuperScript II induces a nucleotide substitution at the BP by reading through the BP 5′-to-2′ phosphodiester bond present in the lariats or lariat-intermediates during reverse transcription, thereby converting the lariat circle into linear cDNA that can be amplified by PCR. Thus, lariat RT-PCR specifies the branched nucleotide from a BPS upstream of the 3′ splice site AG dinucleotide and has been widely used for branch point mapping (Fig. 4a) [31–33]. Lariat RT-PCR products from the wt and mt BRAF RNA splicing (Fig. 3b) were gel-extracted (Fig. 4b) and analyzed by TA-cloning and sequencing (Fig. 4c). After screening of 23 bacterial colonies from each lariat RT-PCR product, we found that eight colonies had an insertion of the wt lariat products and others displayed no insertion. Three adenosines, -29A, -28A and -26A from the intron 8 3′ splice site were identified twice each in the branching reaction of the wt BRAF RNA splicing (Fig. 4c, d). We also identified a single bacterial colony containing a -15A or -25A in wt lariats. For the mt lariat RT-PCR products, we identified 15 colonies with the insertion, of which the -22A, -18A and -15A were identified from multiple colonies for in branching reaction of mt BRAF RNA splicing (Fig. 4c, d) and the -20A and -26A were found each only in a single colony. Surprisingly, none of the screened colonies showed a BP at the -51C (wt) or -51G (mt), nor a cryptic BP at the -88U or -109U [11] as predicted by ESEfinder [34]. The C-to-G mutation at -51 was also found not to alter the conservation value of the predicted 7-nt BPS (Fig. 1b).

In summary, our data demonstrate that wt and mt BRAF RNA select a distinct set of alternative BPs in the intron 8 for splicing, with the wt BRAF using distal BPs (-29A, -28A and -26A) to the intron 3′ splice site for the exon 8^9 splicing and the mt BRAF using proximal BPs (-22A, -18A and -15A) to the intron 3′ splice site for the exon 3^9 splicing (Fig. 4e).

Flexibility or redundancy in BP selection has a role in alternative splicing and was described in both viral [33, 35–37] and human gene expression [27, 38]. Recent genome-wide BP mapping studies indicate that a large proportion of introns have more than one BP, generally clustered in close proximity in relation to the 3′ splice site [27, 38], although a BP could be found in rare case further upstream of a 3′ splice site [27, 38]. Since the predicted -51C [11] from the 3′ splice site of intron 8 identified by ESEfinder [34] or by Human Splice Finder [28] was not mapped as an authentic BP in this study, our data imply that the observed mutations (-435 C-to-A and -51 C-to-G) in the mt BRAF pre-mRNA might disrupt the binding of *trans*-acting factors, such as SRSF6 (SRp55) [11, 39, 40] and SF3b/3a [41–45], to the -51 region and thereby prevent the recruitment of SF1 and U2 snRNA [46–48] to select an authentic distal BP for splicing of BRAF RNA. Consequently, loss of splicing factor binding to the -51 region and activation of a proximal BP usage might lead to skipping of exons 4–8 in splicing of mt BRAF. The minigene system in this report constructed in a classical way [49–52] has some advantage over the minigene in other study [11]. The latter had an extremely large (>1 kb) middle exon (an exon 4/8 fusion exon inserted with a strawberry reporter) and a BRAF exon 9 as a terminal exon fused with a GFP reporter [11]. An oversized internal exon larger than 500 nts has been shown to affect exon definition and thereby RNA splicing [16]. In summary, our observation provides further insight into the molecular mechanisms toward understanding the regulation of alternative splicing of BRAF upon vemurafenib resistance in melanoma.

## Methods

### RT-PCR, in vitro splicing assay and lariat RT-PCR

RT-PCR is performed as described [36] for wt SKMEL-239 cells and C3 SKMEL-239 melanoma cells. Two primer sets were used separately with the primer pair of 3F and 9R for detection of both the constitutive and alternative BRAF RNA splicing and 8F and 9R only for the constitutive exon 8^9 splicing (Additional file 1: Table S1; Fig. 2a). GAPDH RNA was detected with a primer pair described [36] as a loading control.

BRAF pre-mRNAs were prepared by in vitro transcription with T7 RNA polymerase from two-exon, one-intron DNA templates prepared by overlapping PCR [31, 36]. The wt BRAF template has a truncated intron 8 originally from SKMEL-239 cells and the mt BRAF template from C3 SKMEL-239 cells has a chimeric intron 3 and intron 8 of which the intron 3 5′ splice site (64 nts) was fused with the intron 8 3′ splice site (440 nts) including the point mutations in the intron (Fig. 3a). See primer details for template preparation in Additional file 1: Table S1.

In vitro splicing assay was performed as described [29, 36, 53]. Briefly, 4 ng of ^32^P-labeled pre-mRNAs were incubated with HeLa cell nuclear extract at 30 °C for a 2 h in vitro splicing reaction and followed by extraction of splicing products. The splicing products were resolved by electrophoresis on a 6 % denaturing PAGE gel. Autoradiograph was captured by PhosphorImager Storm 860 (GE Healthcare Life Sciences, Pittsburgh, PA).

For lariat RT-PCR [31–33], in vitro splicing products from 100 ng of cold pre-mRNAs were reverse transcribed by Superscript II (Life technologies, Thermo Fisher Scientific) using a primer R and amplified by PCR with a primer pair of R and F1 first followed by a nested primer pair of R and F2 (Fig. 4a; Additional file 1: Table S1). The lariat RT-PCR products were subcloned into the pCR2.1 TOPO vector (Life Technologies) and sequenced.

## Additional file

10.1186/s13578-015-0061-7 BRAF primers used in this study.
